# Adiponectin is associated with cardio-metabolic traits in Mexican children

**DOI:** 10.1038/s41598-019-39801-2

**Published:** 2019-02-28

**Authors:** Juehua He, Carolina Stryjecki, Hudson Reddon, Jesus Peralta-Romero, Roberto Karam-Araujo, Fernando Suarez, Jaime Gomez-Zamudio, Ana Burguete-Garcia, Akram Alyass, Miguel Cruz, David Meyre

**Affiliations:** 1 Department of Health Research Methods, Evidence, and Impact, McMaster University, Hamilton, Canada; 2 Medical Research Unit in Biochemistry, Hospital de Especialidades, Centro Médico Nacional Siglo XXI del Instituto Mexicano del Seguro Social, Mexico City, Mexico; 3 Health Promotion Division, Instituto Mexicano del Seguro Social, Mexico City, Mexico; 4 Centro de investigación sobre enfermedades infecciosas. Instituto Nacional de Salud Pública. Cuernavaca, Morelos, Mexico; 5 Department of Pathology and Molecular Medicine, McMaster University, Hamilton, Canada

## Abstract

The adipocyte-derived adiponectin hormone bridges obesity and its cardio-metabolic complications. Genetic variants at the *ADIPOQ* locus, in *ADIPOR1*, and *ADIPOR2* have been associated with adiponectin concentrations and cardio-metabolic complications in diverse ethnicities. However, no studies have examined these associations in Mexican children. We recruited 1 457 Mexican children from Mexico City. Six genetic variants in or near *ADIPOQ* (rs182052, rs2241766, rs266729, rs822393), *ADIPOR1* (rs10920533), and *ADIPOR2* (rs11061971) were genotyped. Associations between serum adiponectin, genetic variants, and cardio-metabolic traits were assessed using linear and logistic regressions adjusted for age, sex, and recruitment center. Serum adiponectin concentration was negatively associated with body mass index, waist to hip ratio, low-density lipoprotein cholesterol, total cholesterol, triglycerides, fasting glucose, fasting insulin, homeostatic model assessment of insulin resistance, dyslipidemia and overweight/obesity status (7.76 × 10^−40^ ≤ p ≤ 3.00 × 10^−3^). No significant associations between genetic variants in *ADIPOQ*, *ADIPOR1*, and *ADIPOR2* and serum adiponectin concentration were identified (all p ≥ 0.30). No significant associations between the six genetic variants and cardio-metabolic traits were observed after Bonferroni correction (all p < 6.9 × 10^−4^). Our study suggests strong associations between circulating adiponectin concentration and cardio-metabolic traits in Mexican children.

## Introduction

In 2016, the World Health Organization reported that 1.9 billion adults and 381 million children were overweight/obese, resulting in an important global health concern. Obesity is associated with the development of comorbidities (insulin resistance (IR), type 2 diabetes (T2D), dyslipidemia, hypertension), collectively known as the metabolic syndrome^1^. Several therapeutic options are available, however controlling the development of obesity and its resulting complications has proven challenging^2^. Chronic obesity in its more severe forms leads to major reductions in life expectancy, with most of the excess deaths due to heart disease, cancer, and T2D^3^. As a result, obesity imposes a heavy socio-economic burden in both high-income and developing countries^4^.

The Mexican population is a group at high risk for developing obesity and the metabolic syndrome, especially in childhood^5^. The prevalence of obesity in Mexican school-aged children was 11.8% in girls and 17.4% in boys in 2012^5^. The metabolic syndrome prevalence was 9.4% in Mexican adolescents in 2010^6^. The rise of childhood obesity in Mexico is largely explained by a ‘nutritional transition’ which reflects changes in dietary patterns characterized by increased consumption of foods that are high in fat and/or sugar, as well as reduced physical activity^7^. Beyond modifiable factors, the elucidation of biological determinants of obesity and its cardio-metabolic complications is expected to improve prediction, prevention and care, including novel treatments adapted to genetic profiles of high-risk populations^8^.

Adiponectin, an adipocyte-derived secretagogue, may be considered as one of the key hormones bridging obesity and its cardio-metabolic complications^9^. Genetic mouse models have shown that deficiency of adiponectin contributes to IR, while its overexpression in leptin-deficient obese mice promotes adipose tissue expansion and improves insulin sensitivity^10,11^. Adiponectin acts on two receptors (adiponectin receptors 1 and 2) encoded by *ADIPOR1* and *ADIPOR2* genes, both of which appear to show functional redundancy^12^. Simultaneous disruption of both AdipoR1 and AdipoR2 in the liver of leptin-deficient obese mice leads to IR and marked glucose intolerance^12^. In humans, adiponectin is abundantly found in the bloodstream where it makes up 0.01–0.05% of total plasma protein^13^. Low serum adiponectin has been associated with obesity, IR, T2D, dyslipidemia, hypertension and coronary heart disease in cross-sectional studies^9^. Adiponectin level was also negatively associated with incident development of insulin resistance, T2D, dyslipidemia, hypertension, and coronary artery disease^9^. The relationship between adiponectin level and subsequent weight gain has been a topic of interest due to its paradoxical nature, where levels of adiponectin decrease with the development of obesity^14^. Adiponectin was positively associated with weight gain in children, but not in adults in prospective studies^15,16^.

If serum adiponectin levels are influenced by modifiable factors such as physical activity and diet, genetic factors account for 30–93% of variation in adiponectin levels in humans^17,18^. Encoded by the *ADIPOQ* locus and found on chromosome 3q27, adiponectin is a 30 kDA protein with both a collagenous N and a globular C-terminus^18^. Candidate gene studies, and more recently genome-wide association studies (GWAS), fine-mapping or resequencing experiments have identified numerous common and rare variants at the *ADIPOQ* locus associated with serum adiponectin level and metabolic traits^18–24^. If common variants in the *ADIPOR1* and *ADIPOR2* genes have not been associated with serum adiponectin levels, they contribute to IR, T2D and cardiovascular disease risk^18,22,25,26^.

While serum adiponectin levels negatively correlate with obesity, T2D and the components of the metabolic syndrome in Mexican children, high adiponectin concentrations are associated with a metabolically healthy but obese profile in Mexican adults^27–29^. A few studies investigated the association of SNPs in *ADIPOQ ADIPOR1*, and *ADIPOR2* with serum adiponectin levels and cardio-metabolic traits in Mexican and Mexican-American adults^30–33^. However, to date, no study has investigated these genetic associations in Mexican children. This prompted us to analyze in 1 457 Mexican children 1) the association of adiponectin levels with cardio-metabolic traits, 2) the association of six SNPs in *ADIPOQ*, *ADIPOR1*, *ADIPOR2*, and serum adiponectin levels, and 3) the association of the same SNPs with cardio-metabolic traits.

## Results

### Descriptive characteristics of the population

Anthropometric and biochemical characteristics of the 1 457 Mexican children (boys: 52.9%; girls: 47.1%) are summarized in Table 1. The children were 9.24 ± 2.07 years-old and displayed a BMI of 19.65 ± 4.20 kg/m^2^ and a SDS-BMI of 0.71 ± 1.09. Within the sample, 20.8% of children were overweight and 23.0% were obese. Insulin resistance was identified in 11.1% of children, 3.1% had hyperglycemia including one child with T2D. Hypertension and dyslipidemia were present in 1.5% and 34.9% of the sample, respectively. The mean serum adiponectin concentration was 5.26 ± 1.23 µg/mL.

**Table 1:** General characteristics of the studied population of Mexican children.

Trait	Total N = 1 457
Boys/Girls, N (%)	771/686 (52.9/47.1)
Age (years)	9.24 ± 2.07
Adiponectin (µg/ml)	5.26 ± 1.23
BMI (kg/m^2^)	19.65 ± 4.20
SDS-BMI	0.71 ± 1.09
Waist to hip ratio	0.85 ± 0.06
SDS-Waist to hip ratio	2.95 ± 0.33
Systolic blood pressure (mmHg)	98.57 ± 10.86
SDS-Systolic blood pressure	−0.32 ± 1.01
Diastolic blood pressure (mmHg)	66.24 ± 8.80
SDS-Diastolic blood pressure	0.59 ± 0.78
LDL Cholesterol (mg/dL)	102.39 ± 26.42
HDL Cholesterol (mg/dL)	50.60 ± 12.82
Total cholesterol (mg/dL)	157.25 ± 33.56
Triglycerides (mg/dL)	93.62 ± 49.70
Fasting glucose (mmol/L)	4.57 ± 0.53
Fasting insulin (mIU/L)	8.68 ± 7.10
HOMA-IR	1.86 ± 1.52
HOMA-B	36.36 ± 30.36
Hypertension, N (%)	22 (1.5)
Hyperglycemia, N (%)	45 (3.1)
Insulin resistance, N (%)	127 (11.1)
Dyslipidemia, N (%)	509 (34.9)
Underweight, N (%)	30 (2.1)
Normal weight, N (%)	788 (54.1)
Overweight, N (%)	303 (20.8)
Obese, N (%)	335 (23.0)

Abbreviations: BMI, body mass index; HDL, high density lipoprotein cholesterol; HOMA-IR, homeostatic model assessment of insulin resistance; HOMA-B, homeostatic model assessment of beta cell function; LDL, low density lipoprotein cholesterol; SDS, standard deviation scores. Data are means ± standard deviation for continuous traits, and numbers and percentages for categorical traits.

### Association of serum adiponectin concentration with cardio-metabolic traits

We investigated the association of serum adiponectin concentration with cardio-metabolic traits adjusted for sex, age, and recruitment center (Table 2). Serum adiponectin concentration was negatively associated with BMI (β = −0.27 ± 0.02, p = 4.13 × 10^−30^), SDS-BMI (β = −0.33 ± 0.03, p = 6.50 × 10^−32^), WHR (β = −0.18 ± 0.02, p = 8.20 × 10^−12^), SDS-WHR (β = −0.02 ± 3.80 × 10^−3^, p = 2.11 × 10^−10^), LDL cholesterol (β = −0.09 ± 0.02, p = 2.70 × 10^−4^), total cholesterol (β = −0.10 ± 0.02, p = 5.00 × 10^−5^), triglycerides (β = −0.14 ± 0.03, p = 2.98 × 10^−8^), fasting glucose (β = −0.12 ± 0.02, p = 2.00 × 10^−6^), fasting insulin (β = −0.08 ± 0.03, p = 3.00 × 10^−3^), and HOMA-IR (β = −0.10 ± 0.03, p = 3.50 × 10^−4^). Nominal associations (p < 0.05) between serum adiponectin concentration and SBP, DBP, SDS-SBP, SDS-DBP and HOMA-B were observed, but did not reach statistical significance after Bonferroni correction (p > 4.2 × 10^−3^; Table 2). No association between serum adiponectin concentration and HDL cholesterol was observed (p = 0.49).

**Table 2:** Association of serum adiponectin concentrations with cardio-metabolic traits.

Continuous Traits	No additional adjustments	Additional adjustment for BMI
	β ± SE (p-value)	
BMI (kg/m^2^)^a^	**−0.27 ± 0.02 (4.13 × 10** ^**−30**^ **)**	NA
SDS-BMI	**−0.33 ± 0.03 (6.50 × 10** ^**−32**^ **)**	NA
WHR^a^	**−0.18 ± 0.02 (8.20 × 10** ^**−12**^ **)**	−0.02 ± 0.02 (0.43)
SDS-WHR	**−0.02 ± 3.80 × 10** ^**−3**^ **(2.11 × 10** ^**−10**^ **)**	−4.45 × 10^−3^ ± 3.46 × 10^−3^ (0.20)
SBP (mmHg)^a^	−0.07 ± 0.02 (4.00 × 10^−3^)	0.02 ± 0.02 (0.47)
SDS-SBP	−0.06 ± 0.03 (0.03)	0.02 ± 0.03 (0.55)
DBP (mmHg)^a^	−0.07 ± 0.02 (8.00 × 10^−3^)	4.20 × 10^−3^ ± 0.03 (0.87)
SDS-DBP	−0.05 ± 0.02 (0.02)	−8.24 × 10^−3^ ± 0.02 (0.71)
LDL Cholesterol (mg/dL)^a^	**−0.09 ± 0.02 (2.70 × 10** ^**−4**^ **)**	−0.04 ± 0.03 (0.14)
HDL Cholesterol (mg/dL)^a^	0.02 ± 0.02 (0.49)	**−0.09 ± 0.03 (3.17 × 10** ^**−4**^ **)**
Total cholesterol (mg/dL)^a^	**−0.10 ± 0.02 (5.00 × 10** ^**−5**^ **)**	−0.07 ± 0.03 (4.52 × 10^−3^)
Triglycerides (mg/dL)^a^	**−0.14 ± 0.03 (2.98 × 10** ^**−8**^ **)**	−0.02 ± 0.02 (0.47)
Fasting glucose (mmol/L)^a^	**−0.12 ± 0.02 (2.00 × 10** ^**−6**^ **)**	**−0.10 ± 0.02 (4.30 × 10** ^**−5**^ **)**
Fasting insulin (mIU/L)^a^	**−0.08 ± 0.03 (3.00 × 10** ^**−3**^ **)**	0.06 ± 0.02 (0.01)
HOMA-IR^a^	**−0.10 ± 0.03 (3.50 × 10** ^**−4**^ **)**	0.04 ± 0.03 (0.08)
HOMA-B^a^	−0.07 ± 0.03 (0.02)	**0.08 ± 0.02 (1.07 × 10** ^**−3**^ **)**
**Binary Traits**	**OR [95% CI] (p-value)**	
Hypertension	0.75 [0.50-1.13] (0.17)	0.80 [0.53-1.23] (0.31)
Hyperglycemia	0.95 [0.69–1.32] (0.78)	1.03 [0.74–1.45] (0.85)
Insulin resistance	0.83 [0.67–1.02] (0.07)	1.30 [1.01–1.66] (0.04)
Dyslipidemia	**0.75 [0.67–0.84] (1.00 × 10** ^**–6**^ **)**	0.95 [0.94–1.08] (0.42)
Normal weight vs. overweight	**0.39 [0.33–0.46] (2.40 × 10** ^**−26**^ **)**	NA
Normal weight vs. obese	**0.40 [0.34–0.47] (4.84 × 10** ^**−29**^ **)**	NA
Normal weight vs. overweight and obese	**0.41 [0.36–0.47] (7.76 × 10** ^**−40**^ **)**	NA

Abbreviations: BMI, body mass index; DBP, diastolic blood pressure; HDL, high density lipoprotein cholesterol; HOMA-IR, homeostatic model assessment of insulin resistance; HOMA-B, homeostatic model assessment of beta cell function; LDL, low density lipoprotein cholesterol; SBP, systolic blood pressure; SDS, standard deviation scores; WHR, waist-to-hip ratio. Continuous traits: Data presented are β ± SE (p _value_). Data was adjusted for age, sex, and recruitment center. Values in bold indicate significant associations after Bonferroni correction (p < 4.2 × 10^−3^). Binary traits: Data presented are OR [95% CI] (p _value_). Data was adjusted for age, sex, and recruitment center; additional adjustments are for BMI where NA denotes a non-applicable adjustment. Values in bold indicate significant associations after Bonferroni correction (p < 4.2 × 10^−3^). ^a^Inverse normal transformed variables.

When metabolic traits were classified as binary traits (Table 2), serum adiponectin concentration was negatively associated with dyslipidemia (OR = 0.75, 95% CI = 0.67–0.84, p = 1.00 × 10^−6^), normal weight vs. overweight (OR = 0.39, 95% CI = 0.33–0.46, p = 2.40 × 10^−26^), normal weight vs. obese (OR = 0.40, 95% CI = 0.34–0.47, p = 4.84 × 10^−29^), normal weight vs. overweight and obese participants (OR = 0.41, 95% CI = 0.36–0.47, p = 7.76 × 10^−40^). Associations of serum adiponectin concentration with hypertension, hyperglycemia, and IR were not statistically significant (p ≥ 0.07).

We then investigated the association of serum adiponectin concentration with continuous and binary cardio-metabolic traits adjusted for sex, age, recruitment center, and BMI (Table 2). Serum adiponectin concentration was positively associated with HOMA-B (β = 0.08 ± 0.02, p = 1.07 × 10^−3^) and negatively associated with fasting glucose (β = −0.10 ± 0.02, p = 4.30 × 10^−5^) and HDL cholesterol (β = −0.09 ± 0.03, p = 3.17 × 10^−4^). Nominal associations (p < 0.05) between serum adiponectin concentration and fasting insulin, total cholesterol and insulin resistance were observed, but did not reach statistical significance after Bonferroni correction (p > 4.2 × 10^−3^; Table 2). No association was observed for the other traits (p ≥ 0.08).

### Genotype frequency comparison in Mexican children and adults from 1000G for SNPs in/near *ADIPOQ*, *ADIPOR1*, and *ADIPOR2*

Genotype distributions and allele frequencies of the six selected SNPs are presented in Supplementary Table S1. The MAF for *ADIPOQ*, *ADIPOR1*, and *ADIPOR2* SNPs in Mexican children are as follows: 11% for rs10920533, 18% rs2241766, 36% for rs11061971, 38% for rs266729, 43% for rs822393, and 53% for rs182052. Allelic distributions for all selected SNPs were not significantly different from the reported frequencies in the 1000G reference Mexican adult population (p ≥ 0.07).

### Association of SNPs in/near *ADIPOQ*, *ADIPOR1*, and *ADIPOR2* with serum adiponectin concentration

We investigated the association of SNPs in *ADIPOQ* (rs182052, rs2241766, rs266729, rs822393), *ADIPOR1* (rs10920533), and *ADIPOR2* (rs11061971) with serum adiponectin concentration under an additive model, adjusted for sex, age, and recruitment center (Table 3). We did not identify any significant associations between these SNP and serum adiponectin concentrations (all p ≥ 0.30).

**Table 3:** Association of SNPs in/near *ADIPOQ*, *ADIPOR1*, and *ADIPOR2* with serum adiponectin concentration^a^.

SNP		β ± SE	p-value
*ADIPOQ*	rs182052	0.02 ± 0.04	0.60
	rs2241766	−0.01 ± 0.05	0.81
	rs266729	0.01 ± 0.04	0.80
	rs822393	0.03 ± 0.04	0.40
*ADIPOR1*	rs10920533	−0.06 ± 0.60	0.30
*ADIPOR2*	rs11061971	0.04 ± 0.04	0.33

Data presented are β ± SE (p _value_). Data presented follow an additive model, adjusting for age, sex, and recruitment center. ^a^Inverse normal transformed variables.

### Association of SNPs in/near *ADIPOQ*, *ADIPOR1*, and *ADIPOR2* with cardio-metabolic traits

We subsequently tested the association of the aforementioned SNPs with cardio-metabolic traits, adjusted for sex, age, and recruitment center and with and without adjustment for serum adiponectin concentration (Tables 4 and 5). We observed nominal (p < 0.05) associations for *ADIPOR1* rs10920533 with total cholesterol before and after adjusting for serum adiponectin. *ADIPOR1* rs10920533 was also nominally associated with normal weight vs. obese before adjusting for serum adiponectin. We observed nominal associations for *ADIPOR2* rs11061971 with BMI, SDS-BMI, normal weight vs. overweight and obese, and normal weight vs. overweight before and after adjusting for serum adiponectin, and waist circumference before adjustment. *ADIPOQ* rs182052 was nominally associated with waist circumference and SBP before adjusting for serum adiponectin, with BMI and SDS-BMI both before and after adjustment, with normal weight vs. overweight before adjustment, and with normal weight vs. overweight and obese both before and after adjustment. *ADIPOQ* rs266729 was nominally associated with normal weight vs. overweight after adjustment for serum adiponectin. *ADIPOQ* rs822393 was nominally associated with normal weight vs. overweight and obese after adjustment for adiponectin. However, none of the results remained significant after correcting for multiple testing (p < 6.9 × 10^−4^).

**Table 4:** Association of SNPs in/near *ADIPOQ*, *ADIPOR1*, and *ADIPOR2* with continuous metabolic traits.

Continuous traits	Additional adjustment	β ± SE (p-value)					
		rs10920533	rs11061971	rs182052	rs2241766	rs266729	rs822393
BMI (kg/m^2^)	None	0.10 ± 0.06 (0.08)	−0.08 ± 0.04 (0.03)	−0.08 ± 0.03 (0.02)	−0.02 ± 0.0 (0.59)	0.08 × 10^−1^ ± 0.03 (0.82)	0.03 ± 0.03 (0.37)
	Adiponectin	0.08 ± 0.02 (0.14)	**−0.07 ± 0.03 (0.048)**	**−0.07 ± 0.03 (0.02)**	−0.03 ± 0.04 (0.54)	0.01 ± 0.03 (0.77)	0.04 ± 0.03 (0.25)
SDS-BMI	None	0.12 ± 0.07 (0.08)	**−0.11 ± 0.04 (0.01)**	**−0.11 ± 0.04 (4.61 × 10** ^**−3**^ **)**	−0.03 ± 0.05 (0.64)	0.02 ± 0.04 (0.60)	004 ± 0.04 (0.28)
	Adiponectin	0.09 ± 0.06 (0.14)	−0.10 ± 0.04 (0.02)	−0.10 ± 0.04 (6.43 × 10^−3^)	−0.03 ± 0.05 (0.58)	0.02 ± 0.04 (0.53)	0.05 ± 0.04 (0.16)
WHR	None	0.06 ± 0.06 (0.33)	−0.03 ± 0.04 (0.48)	−0.03 ± 0.04 (0.39)	0.04 ± 0.05 (0.44)	−0.08 × 10^−1^ ± 0.04 (0.82)	0.02 ± 0.04 (0.54)
	Adiponectin	0.05 ± 0.06 (0.43)	−0.02 ± 0.04 (0.62)	−0.02 ± 0.04 (0.55)	0.03 ± 0.05 (0.50)	−0.07 × 10^−1^ ± 0.04 (0.84)	0.03 ± 0.04 (0.44)
SDS-WHR	None	4.16 × 10^−3^ ± 8.91 × 10^−3^ (0.64)	−5.67 × 10^−3^ ± 5.70 × 10^−3^ (0.32)	−3.90 × 10^−3^ ± 5.45 × 10^−3^ (0.47)	1.46 × 10^−3^ ± 7.14 × 10^−3^ (0.84)	3.70 × 10^−3^ ± 5.61 × 10^−3^ (0.51)	7.00 × 10^−3^ ± 5.40 × 10^−3^ (0.20)
	Adiponectin	2.27 × 10^−3^ ± 8.81 × 10^−3^ (0.80)	−4.44 × 10^−3^ ± 5.64 × 10^−3^ (0.43)	−2.74 × 10^−4^ ± 5.39 × 10^−3^ (0.61)	9.79 × 10^−4^ ± 7.05 × 10^−3^ (0.89)	4.005 × 10^−3^ ± 5.54 × 10^−3^ (0.46)	8.00 × 10^−3^ ± 5.33 × 10^–3^ (0.13)
SBP (mmHg)	None	0.08 ± 0.06 (0.12)	−0.06 ± 0.04 (0.10)	−0.07 ± 0.03 (4.97 × 10^−2^)	0.03 ± 0.04 (0.54)	−0.03 ± 0.04 (0.38)	−0.01 ± 0.03 (0.71)
	Adiponectin	0.08 ± 0.06 (0.16)	−0.06 ± 0.04 (0.12)	−0.06 ± 0.03 (0.06)	−0.03 ± 0.40 (0.55)	−0.03 ± 0.04 (0.37)	−0.01 ± 0.03 (0.75)
SDS-SBP	None	0.09 ± 0.06 (0.14)	−0.04 ± 004 (0.29)	−0.05 ± 0.04 (0.18)	0.04 ± 0.05 (0.46)	−0.06 ± 0.04 (0.17)	−0.02 ± 0.04 (0.67)
	Adiponectin	0.09 ± 0.06 (0.18)	0.04 ± 0.04 (0.33)	−0.05 ± 0.04 (0.20)	0.04 ± 0.05 (0.47)	−0.06 ± 0.04 (0.17)	−0.01 ± 0.04 (0.71)
DBP (mmHg)	None	0.02 ± 0.06 (0.69)	−0.04 × 10^−1^ ± 0.04 (0.92)	−0.05 ± 0.04 (0.14)	0.03 × 10^−1^ ± 0.05 (0.94)	0.02 ± 0.04 (0.60)	−0.02 ± 0.03 (0.63)
	Adiponectin	0.01 ± 0.06 (0.82)	−0.01 × 10^−1^ ± 0.04 (0.98)	−0.05 ± 0.04 (0.15)	−0.03 × 10^−1^ ± 0.05 (0.94)	0.02 ± 0.04 (0.59)	−0.01 ± 0.03 (0.68)
SDS-SBP	None	−3.03 × 10^−3^ ± 0.05(0.95)	1.10 × 10^−3^ ± 0.03 (0.97)	−0.05 ± 0.03 (0.13)	0.03 ± 0.04 (0.52)	0.01 ± 0.03 (0.78)	−0.04 ± 0.03 (0.20)
	Adiponectin	−0.01 ± 0.05 (0.84)	4.66 × 10^−3^ ± 0.03 (0.88)	−0.05 ± 0.03 (0.14)	0.02 ± 0.04 (0.56)	0.01 ± 0.03 (0.74)	−0.04 ± 0.03 (0.24)
LDL cholesterol (mg/dL)	None	0.11 ± 0.06 (0.05)	0.05 ± 0.04 (0.18)	0.03 × 10^−1^ ± 0.04 (0.93)	0.03 ± 0.05 (0.53)	−0.01 ± 0.04 (0.71)	0.04 × 10^−1^ ± 0.04 (0.90)
	Adiponectin	0.11 ± 0.06 (0.07)	0.06 ± 0.04 (0.12)	−0.01 ± 0.04 (0.79)	0.02 ± 0.05 (0.60)	−0.01 ± 0.04 (0.72)	0.08 × 10^−1^ ± 0.04 (0.83)
HDL cholesterol (mg/dL)	None	0.04 ± 0.06 (0.54)	−0.01 ± 0.04 (0.71)	−0.02 × 10^−1^ ± 0.04 (0.96)	0.02 ± 0.05 (0.61)	−0.06 ± 0.04 (0.10)	−0.03 ± 0.03 (0.47)
	Adiponectin	0.04 ± 0.06 (0.48)	−0.09 × 10^−1^ ± 0.04 (0.80)	0.02 × 10^−1^ ± 0.04 (0.95)	0.02 ± 0.05 (0.68)	−0.06 ± 0.04 (0.09)	−0.02 ± 0.03 (0.48)
Total cholesterol (mg/dL)	None	0.15 ± 0.06 (8.00 × 10^−3^)	0.03 ± 0.04 (0.35)	−0.03 × 10^−1^ ± 0.04 (0.92)	0.04 ± 0.04 (0.39)	−0.04 ± 0.04 (0.22)	−0.01 ± 0.03 (0.72)
	Adiponectin	0.14 ± 0.06 (0.01)	0.04 ± 0.04 (0.23)	0.04 × 10^−1^ ± 0.03 (0.91)	0.03 ± 0.04 (0.46)	−0.04 ± 0.04 (0.22)	−0.09 × 10^−1^ ± 0.03 (0.80)
Triglycerides (mg/dL)	None	0.04 ± 0.06 (0.54)	0.02 ± 0.04 (0.65)	−0.05 ± 0.04 (0.15)	−0.03 ± 0.05 (0.50)	−0.01 ± 0.04 (0.72)	−0.04 × 10^−1^ ± 0.04 (0.90)
	Adiponectin	0.03 ± 0.06 (0.63)	0.02 ± 0.04 (0.55)	−0.05 ± 0.04 (0.18)	−0.03 ± 0.05 (0.52)	−0.01 ± 0.04 (0.72)	−0.02 × 10^−1^ ± 0.04 (0.96)
Fasting glucose (mmol/L)	None	0.04 ± 0.06 (0.49)	0.02 × 10^−1^ ± 0.04 (0.96)	−0.03 ± 0.04 (0.44)	−0.02 ± 0.04 (0.68)	−0.06 ± 0.04 (0.10)	0.02 ± 0.03 (0.58)
	Adiponectin	0.04 ± 0.06 (0.50)	0.08 × 10^−1^ ± 0.04 (0.83)	−0.02 ± 0.03 (0.57)	−0.02 ± 0.04 (0.59)	−0.06 ± 0.04 (0.08)	0.02 ± 0.03 (0.55)
Fasting insulin (mIU/L)	None	0.07 ± 0.06 (0.26)	−0.06 ± 0.04 (0.12)	−0.01 ± 0.04 (0.80)	−0.03 ± 0.05 (0.53)	0.01 ± 0.04 (0.73)	0.05 ± 0.04 (0.21)
	Adiponectin	0.06 ± 0.06 (0.30)	−0.06 ± 0.04 (0.13)	−0.08 × 10^−1^ ± 0.04 (0.84)	−0.04 ± 0.05 (0.47)	0.01 ± 0.04 (0.75)	0.05 ± 0.04 (0.19)
HOMA-IR	None	0.08 ± 0.06 (0.22)	−0.07 ± 0.04 (0.09)	−0.01 ± 0.04 (0.73)	−0.04 ± 0.05 (0.44)	0.05 × 10^−1^ ± 0.04 (0.89)	0.05 ± 0.04 (0.19)
	Adiponectin	0.07 ± 0.06 (0.25)	−0.07 ± 0.04 (0.10)	−0.01 ± 0.04 (0.79)	−0.04 ± 0.05 (0.38)	0.05 × 10^−1^ ± 0.04 (0.91)	0.05 ± 0.04 (0.17)
HOMA-B	None	0.06 ± 0.06 (0.32)	−0.06 ± 0.04 (0.12)	−0.05 × 10^−1^ ± 0.04 (0.90)	−0.03 ± 0.05 (0.53)	0.02 ± 0.04 (0.60)	0.04 ± 0.04 (0.26)
	Adiponectin	0.06 ± 0.06 (0.36)	−0.06 ± 0.04 (0.13)	−0.03 × 10^−1^ ± 0.04 (0.93)	−0.04 ± 0.05 (0.47)	0.02 ± 0.04 (0.62)	0.04 ± 0.04(0.24)

Abbreviations: BMI, body mass index; DBP, diastolic blood pressure; HDL, high density lipoprotein cholesterol; HOMA-IR, homeostatic model assessment of insulin resistance; HOMA-B, homeostatic model assessment of beta cell function; LDL, low density lipoprotein cholesterol; SBP, systolic blood pressure; SDS, standard deviation scores; WHR, waist-to-hip ratio. Continuous traits: Data presented are β ± SE (p _value_). All models were adjusted for age, sex, and recruitment center.

**Table 5:** Association of SNPs in/near *ADIPOQ*, *ADIPOR1*, and *ADIPOR2* with binary metabolic traits.

Binary traits	Additional adjustment	OR [95% CI] (p-value)					
		rs10920533	rs11061971	rs182052	rs2241766	rs266729	rs822393
Hypertension	None	1.37 [0.54–3.50] (0.51)	1.56 [0.83–2.92] (0.17)	0.76 [0.42–1.40] (0.38)	0.95 [0.43–2.10] (0.91)	0.77 [0.40–1.46] (0.43)	0.53 [0.28–1.03] (0.06)
	Adiponectin	1.35 [0.53–3.44] (0.53)	1.57 [0.84–2.93] (0.16)	0.77 [0.42–1.42] (0.40)	0.98 [0.44–2.16] (0.95)	0.77 [0.40–1.48] (0.44)	0.54 [0.28–1.04] (0.07)
Hyperglycemia	None	1.04 [0.51–2.10] (0.92)	0.77 [0.49–1.23] (0.28)	1.02 [0.66–1.57] (0.93)	0.92 [0.52–1.62] (0.76)	0.67 [0.42–1.07] (0.09)	0.94 [0.62–1.44] (0.79)
	Adiponectin	1.04 [0.52–2.12] (0.90)	0.77 [0.48–1.22] (0.26)	1.02 [0.66–1.57] (0.94)	0.92 [0.52–1.62] (0.77)	0.66 [0.41–1.06] (0.09)	0.94 [0.62–1.43] (0.76)
Insulin resistance	None	1.28 [0.84–1.94] (0.24)	0.94 [0.70–1.26] (0.68)	0.94 [0.71–1.23] (0.64)	0.85 [0.59–1.22] (0.38)	1.04 [0.79–1.36] (0.80)	1.09 [0.84–1.43] (0.52)
	Adiponectin	1.26 [0.83–1.91] (0.28)	0.94 [0.70–1.26] (0.69)	0.94 [0.72–1.24] (0.68)	0.84 [0.59–1.21] (0.36)	1.04 [0.79–1.37] (0.78)	1.10 [0.84–1.44] (0.48)
Dyslipidemia	None	1.12 [0.87–1.44] (0.38)	0.90 [0.77–1.06] (0.22)	0.89 [0.76–1.04] (0.15)	1.03 [0.84–1.26] (0.77)	1.04 [0.89–1.22] (0.61)	1.00 [0.86–1.17] (1.00)
	Adiponectin	1.10 [0.85–1.41] (0.47)	0.92 [0.78–1.08] (0.31)	0.90 [0.77–1.06] (0.21)	1.02 [0.83–1.25] (0.84)	1.04 [0.89–1.23] (0.60)	1.01 [0.86–1.18] (0.92)
Normal weight vs. Overweight	None	0.91 [0.65–1.26] (0.57)	0.75 [0.61–0.92] (6.00 × 10^−3^)	0.77 [0.63–0.92] (6.00 × 10^−3^)	1.03 [0.80 −1.31] (0.84)	1.21 [0.99–1.48] (0.06)	1.09 [0.90–1.32] (0.35)
	Adiponectin	0.92 [0.65–1.30] (0.64)	0.74 [0.59–0.92] (7.00 × 10^−3^)	0.78 [0.63–0.95] (0.01)	0.97 [0.74–1.26] (0.80)	1.25 [1.01–1.55] (0.04)	1.13 [0.92–1.38] (0.24)
Normal weight vs. Obese	None	1.42 [1.08–1.87] (0.01)	0.85 [0.71–1.02] (0.09)	0.90 [0.75–1.07] (0.24)	0.92 [0.73–1.15] (0.46)	1.08 [0.90–1.28] (0.43)	1.13 [0.95–1.34] (0.15)
	Adiponectin	1.27 [0.95–1.71] (0.10)	0.87 [0.71–1.06] (0.15)	0.90 [0.74–1.09] (0.27)	0.91 [0.71–1.17] (0.47)	1.10 [0.90–1.33] (0.35)	1.20 [1.00–1.44] (0.05)
Normal weight vs. Overweight and Obese	None	1.17 [0.92–1.49] (0.19)	0.80 [0.69–0.94] (6.00 × 10^−3^)	0.84 [0.72–0.97] (0.02)	0.96 [0.79–1.16] (0.68)	1.14 [0.98–1.32] (0.10)	1.12 [0.97–1.30] (0.13)
	Adiponectin	1.12 [0.86–1.45] (0.39)	0.80 [0.68 −0.95] (9.00 × 10^−3^)	0.83 [0.71–0.98] (0.02)	0.94 [0.76–1.16] (0.57)	1.16 [0.98–1.37] (0.08)	1.17 [1.00–1.38] (4.60 × 10^−2^)

Data presented are OR [95% CI] (p _value_). All models were adjusted for age, sex, and recruitment center.

### Statistical power calculations

Statistical power calculations are summarized in Supplementary Tables S2–S6. Using a sample of 1 457 participants, our study had at least 80% power to detect effect sizes/beta values of 0.2 or greater for associations between serum adiponectin and SNPs with MAF of 0.2 or greater for p-value = 8.3 × 10^−3^.

For associations between serum adiponectin and continuous cardio-metabolic traits, we conducted an example statistical power calculation for the association of serum adiponectin and SBP, for which we had at least 80% power to detect beta values of 0.9 or greater for p-value = 4.2 × 10^−3^. For associations between serum adiponectin and binary cardio-metabolic traits, we conducted an example statistical power calculation for the association of serum adiponectin and insulin resistance. With 127 cases in a sample of 1 457 participants, we had at least 80% power to detect effect sizes/odds ratios of 1.40 or greater for p-value = 4.2 × 10^−3^.

For associations between the six SNPs and continuous cardio-metabolic traits, we also examined the association between SNPs and SBP, for which we had at least 80% power to detect beta values of 2.5 or greater when MAF is 0.2 or greater for p-value = 6.9 × 10^−4^. For associations between the six SNPs and binary cardio-metabolic traits, we also examined the association between SNPs and insulin resistance, for which we had at least 80% power to detect effect sizes/odds ratios of 1.3 or greater when MAF is 0.1 or greater for p-value = 6.9 × 10^−4^.

## Discussion

In the present study, we assessed the relationship between serum adiponectin concentration and cardio-metabolic traits and the association of 6 SNPs in *ADIPOQ*, *ADIPOR1*, and *ADIPOR2* with adiponectin serum levels and cardio-metabolic traits in Mexican children. We also compared the SNP genotypic distributions between Mexican children and adults from the 1000G. We found strong negative associations for adiponectin levels with BMI, WHR, LDL cholesterol, total cholesterol, triglycerides, fasting glucose, fasting insulin, and HOMA-IR, as well as dyslipidemia, overweight and obesity status. Further adjustment for BMI removed most of these associations, to the exception of fasting glucose. The same adjustment resulted in significant association between serum adiponectin concentration, HDL cholesterol and HOMA-B. The 6 SNPs had similar genotypic distribution in Mexican children and adults. We did not find any association between these SNPs and serum adiponectin concentration. While nominal associations were found between *ADIPOR1* rs10920533, *ADIPOR2* rs11061971, and *ADIPOQ* rs182052 and cardio-metabolic traits, none remained significant after Bonferroni correction for multiple testing. Based on our statistical power calculations, our study was only modestly powered (Supplementary Tables S2–S6), and lack of associations may be confirmed in larger samples.

The Mexican population is at high risk for developing obesity, IR, dyslipidemia and T2D due to genetic predisposition in combination with recent demographic, socioeconomic and nutrition transitions^34–41^. Reduced physical activity due to urbanization, together with shifts in dietary patterns away from traditional high-fiber foods in favor of processed foods have resulted in the rise of non-communicable chronic diseases among all age groups^42^. In 2011, the prevalence of overweight and obesity in Mexican children reached 34.4%, representing one of the highest rates in the world^43^. In our sample, the prevalence of overweight/obesity exceeded the national average (43.8%), possibly due to our strategy to recruit children within an urban setting. The prevalence of hypertension in our sample (1.5%) was lower than previously reported (4.7% to 14%)^42,44,45^; however, previous studies classify hypertension using percentiles rather than a threshold, making comparisons difficult. The prevalence of IR in our sample (11%) was also low. A cross-sectional study of Mexican children aged 7–18 estimated the prevalence of IR at 20.3%, while the National Health and Nutrition Examination Survey found 52.1% of obese Mexican-Americans aged 12–19 to have IR^46^. The gradual increase of insulin and glucose concentrations observed during adolescence may partially explain this discrepancy^47,48^. We report an exceptionally high prevalence of dyslipidemia in our sample (34.9%). While this high prevalence may be reflective of a diet rich in refined carbohydrates and animal fats but limited in fiber, we cannot exclude the possibility that it may stem from the employed definition of dyslipidemia within our study^49^. Dyslipidemia is routinely defined by abnormal concentrations of one or two lipids, however we used three lipids, thereby artificially increasing the prevalence of dyslipidemia in our sample. The mean serum adiponectin concentration in our sample was lower than in previous reports in Mexican children^27,29^. Differences in the prevalence of obesity, blood samples (i.e. serum vs. plasma) and laboratory tests (i.e. radioimmunoassay vs enzyme immunoassay) can significantly affect measured adiponectin concentrations, making comparisons challenging.

Adiponectin is an insulin-sensitizing hormone secreted from the adipose tissue and is negatively associated with obesity and T2D in epidemiological studies^50^. Adiponectin plays an important role in modulating glucose and lipid metabolism by activating AMP-dependent kinase signaling^51^. The relationship between low serum adiponectin and obesity, IR, T2D, dyslipidemia, hypertension, and cardio-vascular disease has been extensively studied in adults^50^. Adiponectin levels have been found to be lower in obese European and East Asian children^52,53^. Here, we extended the negative association between serum adiponectin level and childhood overweight/obesity status to the Mexican population. The associations between serum adiponectin and continuous cardio-metabolic traits have been previously investigated in Mexican children in modestly sized studies. Consistent with our results, Cruz *et al*. determined negative associations with plasma adiponectin and BMI, insulin concentrations and HOMA-IR in an independent sample^29^. More recently, plasma adiponectin was inversely associated with insulin concentrations, TG, and HOMA-IR in obese Mexican children with the metabolic syndrome^27^. Our results evidenced an inverse association with adiponectin and WHR, LDL-C, total cholesterol, and fasting glucose, which has previously been shown in Latino and Hispanic youth, but not in a Mexican population^54,55^. We also observed an inverse association with adiponectin and dyslipidemia which is consistent with previous reports in a multiethnic adult population and European children^56,57^. Further adjustment for BMI substantially modified the pattern of association between serum adiponectin and cardio-metabolic traits, confirming that adiponectin has an important role in the regulation of body weight^22,58^. Taken together, our results suggest that adiponectin levels may contribute to the link between obesity, IR, glucose homeostasis, and dyslipidemia at a young age.

Several common and rare variants at the *ADIPOQ* locus appear to cause substantial changes in circulating adiponectin concentrations^18,59^. The most frequently studied *ADIPOQ* variants associated with altered adiponectin concentrations include rs17300539, rs266729, rs2241766, and rs1501299^18^. The rs17300539 variant is strongly associated with increased circulating adiponectin due to enhanced *ADIPOQ* promoter activity^60^. Associations with rs266729 and serum adiponectin are inconsistent, however the general trend suggests a decrease in adiponectin concentration which is further evidenced by lower *ADIPOQ* promoter activity^60^. *ADIPOQ* rs2241766 is strongly associated with lower adiponectin levels, possibly due to differences in RNA splicing or stability and rs1501299 is mainly associated with lower adiponectin levels^60^.

Associations with *ADIPOQ* variants and adiponectin levels have been investigated in various populations, however limited information exists in the Mexican population^60^. *ADIPOQ* rs17300539 was associated with higher adiponectin concentrations in a study of 1 153 Hispanic Americans from San Antonio^61^. In a cross-sectional study of 242 Mexican-Mestizo adults, a positive association with *ADIPOQ* rs1501299 and circulating adiponectin was observed^31^. In the present study, we did not identify any significant associations with the selected *ADIPOQ* SNPs and serum adiponectin concentration, possibly due to limited power, age- or ethnic-dependent effects. To our knowledge, this is the first study to examine the association of genetic variants in *ADIPOQ* with serum adiponectin levels in a pediatric Mexican population. Further investigation with a more exhaustive SNP selection and larger sample sizes is warranted.

We subsequently tested the associations of *ADIPOQ* SNPs and cardio-metabolic traits and found nominally significant inverse associations between rs182052 and BMI and obesity status. The association of *ADIPOQ* rs182052 with BMI is consistent with findings by Sutton *et al*. who found the A allele of rs182052 associated with lower BMI in 811 Hispanic adults from San Antonio^62^. However, Richardson *et al*. found a positive association with the G allele of *ADIPOQ* rs182052 and BMI in 439 Mexican American adults from San Antonio and a trend for increased obesity risk has been observed in a small sample of Mexican children^33,63^. Among Brazilians, the A allele of *ADIPOQ* rs182052 was associated with a greater BMI and risk of obesity^64^. However, studies in European adult populations were unable to identify associations with the A allele of *ADIPOQ* rs182052 and BMI^65,66^. These results suggest possible age-dependent associations of *ADIPOQ* SNPs in children with BMI which may be considered in future replication studies in Mexican children.

We did not observe an association between genetic variants in *ADIPOR1* and *ADIPOR2* and serum adiponectin which is consistent with previous studies. GWAS in diverse ethnic groups did not identify *ADIPOR1* or *ADIPOR2* loci as important contributors to serum adiponectin level variation^22,67,68^. Cohen *et al*. investigated the association of *ADIPOR1* and *ADIPOR2* with serum adiponectin levels in Caucasian and African-American women but failed to show an association^69^. Subsequently, Matther *et al*. did not find associations with *ADIPOR1* and *ADIPOR2* and serum adiponectin levels in the Diabetes Prevention Program^26^. More recently, a meta-analysis of 2 355 European-Australians failed to find an association with serum adiponectin and genetic variants in adiponectin receptors^70^. We studied these associations in a Mexican population for the first time and our results are in line with previous publications. We also identified nominally significant associations between *ADIPOR1* rs10920533 and total cholesterol and *ADIPOR2* rs11061971 and obesity risk. Very few studies have examined genetic variation in *ADIPOR1* and *ADIPOR2* in relation to these cardio-metabolic traits, making comparisons challenging. Previous work in adult European populations suggests associations with *ADIPOR1* rs10920533 and *ADIPOR2* rs11061971 and IR, but we were unable to confirm these associations in the present pediatric Mexican population^71^. Further investigation is needed to determine the validity of these associations.

Despite the strong association between adiponectin levels and cardio-metabolic traits, we failed to identify associations with the selected SNPs and cardio-metabolic traits after Bonferroni correction. A possible explanation is that the association between adiponectin and metabolic traits is not causal and can be explained by confounding. Observational epidemiology is prone to confounding, reverse causation, and other sources of bias, thus our results should be interpreted with caution. Adiponectin concentration is inversely associated with obesity and T2D, however it is not yet known whether altered adiponectin concentrations are causal or merely a disease marker. Combining genetic epidemiology with classic epidemiology is one way to strengthen causality. For example, the common *ADIPOQ* variant, rs266729 alters ADIPOQ gene expression and has consistently been associated with lower serum adiponectin concentrations and increased risk of IR and T2D^19,50,72^. Future work in the Mexican population including GWAS for adiponectin levels and Mendelian randomization studies are needed to determine the causal links between this hormone and the development of cardio-metabolic diseases.

Our study has several strengths. It is the first to investigate the association of genetic variation in *ADIPOQ*, *ADIPOR1* and *ADIPOR2*, adiponectin concentrations, and cardio-metabolic traits in a pediatric Mexican population. Measures of serum adiponectin concentration were available, allowing us to investigate the effects of genetic variants on adiponectin levels in addition to diverse cardio-metabolic traits. Furthermore, our study combines classic and genetic epidemiology to strengthen our conclusions. Children represent a purer phenotype as they have less exposure duration to an obesogenic environment, relative to adults^58^. Studying these associations in children may therefore provide more insight into the early biological determinants of obesity and cardio-metabolic complications. Limitations include the selection of *ADIPOQ*, *ADIPOR1*, and *ADIPOR2* SNPs which was not exhaustive and did not include more recent GWAS discoveries^22,73^. Our study is also modestly powered to identify genetic effects, especially after adjusting for multiple testing correction^74^. Study participants were randomly selected from Mexico City and is therefore representative of the urban population of central Mexico, not of the Mexican population as a whole. Furthermore, the Mexican population is admixed with Native American, European, and West African ancestries with proportions varying within different regions of the country. Because we did not have ancestry-informative markers, we could not adjust for potential population stratification. Also, due to the cross-sectional nature of this study, causality cannot be inferred from the associations between serum adiponectin level and cardio-metabolic traits. Some cardio-metabolic traits are also correlated with each other (Supplementary Table S7), making it difficult to discern whether associations between serum adiponectin and cardio-metabolic traits are direct or indirect, and may be mediated by certain outcomes. However, past Mendelian randomization studies have shown that various cardio-metabolic traits, such as HOMA-IR, have a causal relationship with circulating adiponectin levels^9^. Furthermore, past studies have also identified cardio-metabolic traits, including BMI, WHR, fasting insulin, triglycerides, and HDL-cholesterol, that are affected by genetic determinants of adiponectin levels^22^. These studies support the idea that these cardio-metabolic traits are largely and often found to be associated with adiponectin levels, thus the possibility of confounding is very difficult to accurately discern and control for.

In conclusion, our study suggests strong associations between serum adiponectin level and cardio-metabolic traits in a young Mexican population. Further well-powered studies are needed to elucidate the causal relationship between genetic variation in *ADIPOQ*, *ADIPOR1* and *ADIPOR2*, serum adiponectin level, and development of cardio-metabolic complications.

## Methods

### Study population

A total of 1 559 children between the ages of 5 and 17 were randomly selected to participate in a cross-sectional study from four areas in Mexico City at the Primary Care Unit of the National Mexican Social Security Institute (Cuauhtémoc West, Independencia South, Nezahualcóyotl Est and Morelos North area). Recruitment was done in collaboration with local public schools. The study started in July 2011 and is still ongoing. A trained pediatrician performed all the anthropometric measurements. Blood samples were collected for biochemical measurements and DNA extraction. Children who had diagnosis of infectious disease, gastrointestinal disorders, administration of antimicrobial agents (within 6 months prior to study), incomplete questionnaires or biological samples were excluded. The child’s assent and written informed consent from the parents/guardians was obtained prior to enrolment into the study. The study protocol was approved by the Mexican Social Security Institute National Committee and the Ethical Committee Board. All procedures were conducted in accordance with the relevant guidelines and regulations of the Declaration of Helsinki^75^.

### Phenotyping

All participants were weighed using a digital scale (Seca, Hamburg, Germany) and height was measured with a portable stadiometer (Seca 225, Hamburg, Germany). Height, weight and body mass index (BMI), calculated as weight (kg)/height (m)^2^, were converted to age- and gender- adjusted standard deviation scores (SDS-Height, SDS-Weight and SDS-BMI, respectively) using the LMS method according to guidelines from the Centers for Disease Control (CDC)^76,77^. Waist circumference (WC) was measured at the midpoint between the lowest rib and the iliac crest after a normal exhalation with children in the standing position. Hip circumference was measured at the level of the greater trochanters. The waist to hip ratio (WHR) was also converted to age- and gender- adjusted standard deviation scores (SDS-WHR) using the LMS method and growth charts based on US National Health and Nutrition Survey, cycle III (NHANES III)^78^. BMI was used to classify children as underweight, normal weight, overweight, or obese, according to the Centers for Disease Control and Prevention CDC 2000 references. Systolic and diastolic blood pressure (SBP and DBP) were measured using a mercurial sphygmomanometer (ALPK2, Tokyo, Japan). Blood pressure readings were taken for each participant twice on the right arm in a sitting position with 5 minutes rest between each measurement and the mean of the two readings was determined. Age- and gender- adjusted standard deviations scores for SBP and DBP (SDS-SBP and SDS-DBP) were calculated using methods specified by the fourth report from the National High Blood Pressure Education Program (NHBPEP) in children and adolescents^79^. Hypertension was defined as average measured blood pressure above the American Heart Association’s recommendations (systolic ≥ 140 mmHg or diastolic ≥ 90 mmHg). Blood samples were obtained following a 12 hour fast and were analyzed for glucose, total cholesterol (TC), high-density lipoprotein cholesterol (HDL-C), low-density lipoprotein cholesterol (LDL-C), and triglycerides (TG) using the ILab 350 Clinical Chemistry System (Instrumentation Laboratory IL. Barcelona Spain). Dyslipidemia was defined as fasting TG ≥ 100 mg/dL (0–9 years of age) or TG ≥ 130 mg/dL (10–19 years of age) and/or HDL-C < 35 mg/dL and/or LDL-C ≥ 130 mg/dL, according to current recommendations^80,81^. Insulin (IU) was measured by chemiluminescence (IMMULITE, Siemens, USA) and homeostatic model assessment of insulin resistance (HOMA-IR) and beta-cell function (HOMA-B) were calculated using the equation by Matthews *et al*.^82^. Due to the risk of blood hemolysis, fasting insulin values < 1 µU/mL were discarded from the study. Insulin resistance was defined as HOMA-IR ≥ 3.4 (the 90^th^ percentile of HOMA-IR in a population of healthy Mexican children)^83^. The 2003 ADA criteria for FPG were used to classify participants as having normal glucose tolerance (NGT), impaired fasting glucose (IFG), or T2D. In absence of oral glucose tolerance test (OGTT) 2-hour fasting plasma glucose value, we used the 2003 American Diabetes Association criteria to define normal fasting glucose (NFG, FPG ≤ 5.6 mmol/L), impaired fasting glucose (IFG, FPG of 5.6 to 6.9 mmol/L), and T2D (FPG ≥ 7.0 mmol/L), as previously described^84,85^. Hyperglycemia was defined as FPG > 5.6 mmol/L. Total adiponectin (µg/mL) was determined by ELISA (Human Adiponectin ELISA Kit, Millipore, St. Charles, MO, USA).

### DNA extraction, SNP selection, and genotyping

Genomic DNA was isolated from peripheral blood using a standard extraction protocol on an Autogen FLEX STAR (Holliston, Massachusetts USA). We selected 10 SNPs in *ADIPOQ* (rs2241766, rs266729, rs822393, rs17366568, rs182052, rs4632532, rs7649121), *ADIPOR1* (rs10920533), and *ADIPOR2* (rs11061971, rs16928751) associated with cardio-metabolic traits in literature and harboring minor allele frequencies ≥10% in the Mexican population according to the HapMap database. Genotyping of the SNPs was performed using the TaqMan Open Array Real-Time PCR System (Life Technologies, Carlsbad, USA), following the manufacturer’s instructions. Three SNPs (rs4632532, rs7649121, rs16928751) did not reach valid Open Array assay scores. The Open Array experiment involved 64 polymorphisms in total. From the initial sample of 1 559 participants, 102 were excluded from the current analysis because (i) no blood sample was collected for DNA extraction; (ii) DNA extraction was unsuccessful; (iii) the individual genotyping success rate of the Open Array experiment based on the 64 polymorphisms was <90.6% 6 genotypes missing). The current analysis included 1 457 children with both genotypic and clinical data available. Only one SNP out of seven did not pass the quality control criteria (rs17366568). The six remaining SNPs harbored a genotyping call rate between 97 and 99%, and no deviation from Hardy-Weinberg equilibrium was observed (p between 0.35 and 0.97; Supplementary Table S1). For quality control purposes, we also compared allele frequencies in our sample with adult Mexican-American reference populations in the 1000 Genomes Project (1000G; Supplementary Table S1). Allele frequencies in our study were not significantly different from the reported frequencies in the 1000G for all SNPs (Supplementary Table S1).

### Statistical analyses

The statistical analyses were conducted using the SPSS software (version 20.0) or R (version 3.1.2). We followed the strategy reported previously by Ronald J Feise and considered independent Bonferroni corrections for each question asked^86^. For associations of serum adiponectin with cardio-metabolic traits, two-tailed p-values < 4.2 × 10^−3^ after Bonferroni correction (0.05/12) were considered statistically significant. For association of SNPs in *ADIPOQ*, *ADIPOR1*, and *ADIPOR2* with serum adiponectin concentration, p-values < 8.3 × 10^−3^ (0.05/6) were considered statistically significant. For association of the same SNPs with quantitative traits, p-values < 6.9 × 10^−4^ (0.05/72) were considered statistically significant. QUANTO software was used for statistical power calculations, assuming normal distribution of quantitative traits, 80% power, and using p-values adjusted for multiple comparisons. Non-biological outlier data were discarded using a Cook’s distance test followed by an expert verification. Based on Shapiro-Wilk test (Supplementary Table S8), all the untransformed traits of interest deviated significantly from normality. Hence, rank based inverse normal transformations were applied wherever substantial deviations from normality were observed (Supplementary Fig. S1). Differences in *ADIPOQ*, *ADIPOR1*, and *ADIPOR2* SNP allele frequencies were determined by a Chi-square test. Multiple linear and logistic regressions were used to assess associations, while adjusting for covariates of age, sex, and recruitment center. Additional adjustments for BMI or serum adiponectin level were performed for associations with cardio-metabolic traits to investigate the mediation effect of these intermediate traits. An interaction term for Pearson’s correlation coefficients and associated p-values were found between all continuous cardio-metabolic traits (Supplementary Table S7). An additive model was used in all the genetic analyses. The minor allele was considered as the effect allele.

## Supplementary information

Supplementary online material

## Data Availability

The dataset generated during and/or analyzed during the current study are available from the corresponding author on reasonable request.
