# Supplementary material for: Adiponectin is associated with cardio-metabolic traits in Mexican children
Source: Sci Rep. 2019 Feb 28;9:3084. doi: 10.1038/s41598-019-39801-2 (PMC6395686; doi:10.1038/s41598-019-39801-2)
Supplement: Supplementary file 1 — Supplementary online material [file 41598_2019_39801_MOESM1_ESM.pdf]

Adiponectin is associated with cardio-metabolic traits in Mexican children

Juehua He[1,a], Carolina Stryjecki[1,a], Hudson Reddon[1], Jesus Peralta-Romero[2], Roberto Karam-Araujo[3], Fernando Suarez[2], Jaime Gomez-Zamudio[2], Ana Burguete-Garcia[4], Akram Alyass[1], Miguel Cruz[2,*], David Meyre[1,5,*]

[a]These authors contributed equally to this work

[1]Department of Health Research Methods, Evidence, and Impact, McMaster University, Hamilton, Canada

[2]Medical Research Unit in Biochemistry, Hospital de Especialidades, Centro Médico Nacional Siglo XXI del Instituto Mexicano del Seguro Social, Mexico City, Mexico

[3]Health Promotion Division, Instituto Mexicano del Seguro Social, Mexico City, Mexico

[4]Centro de investigación sobre enfermedades infecciosas. Instituto Nacional de Salud Pública. Cuernavaca, Morelos, Mexico

[5]Department of Pathology and Molecular Medicine, McMaster University, Hamilton, Canada

Corresponding authors*:

Dr. David Meyre. Department of Clinical Epidemiology and Biostatistics, McMaster University, Room 3205, Michael DeGroote Centre for Learning & Discovery, 1280 Main Street West, Hamilton, ON L8S 4K1, Canada. Tel: 905.525.9140 Ext. 26802. Fax: 905.528.2814. Email: meyred@mcmaster.ca. Dr. Miguel Cruz, Unidad de Investigación Médica en Bioquímica, Hospital de Especialidades Centro Médico Nacional Siglo XXI, Instituto Mexicano del Seguro Social, Av. Cuauhtémoc, 330 C.P. 06725, México, D.F. Tel: 52 55 57612358; Fax: 5255 56276914. Email: mcruzl@yahoo.com

Supplementary Table S1: Description of the six adiponectin SNPs studied

SNP (*Gene*)	Major / Minor Allele	Mexican children allele count		1000 Genomes allele count		1000G MAF	Study MAF	Call Rate	HWE P-value	Allele count Chi^2 (P-value)
rs10920533 (*ADIOPR1*)	G / A	G	A	G	A	0.11	0.11	0.966	0.35	0.02 (0.89)
		2519	297	114	14					
rs11061971 (*ADIPOR2*)	T / A	T	A	T	A	0.42	0.36	0.982	0.97	2.01 (0.16)
		1831	1031	74	54					
rs182052 (*ADIPOQ*)	G / A	G	A	G	A	0.47	0.53	0.984	0.76	2.11 (0.15)
		1336	1532	68	60					
rs2241766 (*ADIPOQ*)	T / G	T	G	T	G	0.14	0.18	0.972	0.67	1.61 (0.21)
		2310	524	110	18					
rs266729 (*ADIPOQ*)	C / G	C	G	C	G	0.30	0.38	0.989	0.61	3.22 (0.07)
		1776	1104	89	39					
rs822393 (*ADIPOQ*)	C / T	C	T	C	T	0.41	0.43	0.986	0.38	1.43 (0.23)
		1529	1345	75	53					

Abbreviations, HWE, Hardy-Weinberg equilibrium; MAF, minor allele frequency; SNP, single nucleotide polymorphism. Pearson's Chi^2 test was used to compare the allele counts of our study with the reference counts from 1000G.

Supplementary Table S2: Sample sizes needed to detect significant association between serum adiponectin and the six SNPs with a power of 80% and a two-sided p-value of 8.3 x 10^{-3} (adjusted for multiple testing) by beta coefficient and allele frequency for risk allele

	**Minor allele frequency**						
**Allelic beta**	0.01	0.05	0.1	0.2	0.3	0.4	0.5
0.10	92594	19294	10180	5724	4359	3814	3661
0.20	23144	4819	2540	1426	1085	949	911
0.30	10283	2138	1126	631	479	418	401
0.40	5781	1200	631	352	267	233	223
0.50	3698	766	401	223	168	147	141
0.60	2566	530	277	153	115	100	96
0.70	1884	388	202	111	83	72	69
0.80	1441	295	153	83	62	53	51
0.90	1137	232	120	64	48	41	39
1.00	920	187	96	51	37	32	30
1.50	405	80	39	19	12	10	9
2.00	225	42	19	6	< 1	< 1	< 1
2.50	142	24	9	< 1	< 1	< 1	< 1
3.00	97	15	< 1	< 1	< 1	< 1	< 1
3.50	69	8	< 1	< 1	< 1	< 1	< 1

Abbreviations: SBP, systolic blood pressure; SNP: single nucleotide polymorphism. Calculations are based on serum adiponectin mean 5.26 and standard deviation 1.23.

Supplementary Table S3: Sample sizes needed in a cohort design to detect significant association between serum adiponectin and SBP across a range of beta coefficients with statistical power of 80% and two-sided p-values of 0.05 (unadjusted) and 4.2×10^{-3} (adjusted for multiple testing)

β	P=0.05	P=4.2×10^{-3}
0.10	61183	107112
0.20	15293	26773
0.30	6795	11895
0.40	3820	6688
0.50	2444	4278
0.60	1696	2969
0.70	1245	2179
0.80	952	1667
0.90	751	1316
1.00	608	1064
1.10	502	878
1.20	421	737
1.30	358	627
1.40	308	540
1.50	268	469

Abbreviation: SBP, systolic blood pressure. Calculations are based on serum adiponectin standard deviation 1.23, SBP mean 98.57 and standard deviation 10.86.

Supplementary Table S4: Number of cases per 10 controls to detect significant association between serum adiponectin and IR across a range of beta coefficients with statistical power of 80% and two-sided p-values of 0.05 (unadjusted) and 4.2 x 10^{-3} (adjusted for multiple testing)

OR	P=0.05	P=4.2 x 10^{-3}
1.10	629	1102
1.20	173	303
1.30	84	147
1.40	52	91
1.50	36	63
1.60	27	48
1.70	22	38
1.80	18	31
1.90	15	27
2.00	13	23
2.10	12	21
2.20	11	19
2.30	10	17
2.40	9	16
2.50	8	15

Abbreviation: IR, insulin resistance. Calculations are based on serum adiponectin standard deviation 1.23, and 11% baseline risk for IR.

Supplementary Table S5: Sample sizes needed in a cohort design to detect significant association between the six SNPs and SBP with statistical power of 80% and two-sided p-values of 0.05 (unadjusted) and 6.9 x 10^{-4} (adjusted for multiple testing) across a range of beta coefficients and allele frequencies for risk allele

**β**	**P=0.05**					**P=6.9 x 10^{-4}**				
	**Minor allele frequency**					**Minor allele frequency**				
	0.05	0.10	0.20	0.40	0.50	0.05	0.10	0.20	0.40	0.50
0.10	974411	514270	289275	192849	185135	>1000000	>1000000	660515	440340	422726
0.20	243600	128565	72316	48209	46281	556222	293557	165122	110078	105675
0.30	108264	57138	32138	21424	20567	247205	130465	73383	48919	46962
0.40	60897	32138	18076	12049	11567	139049	73383	41274	27513	26412
0.50	38973	20567	11567	7710	7402	88988	46962	26412	17605	16900
0.60	27063	14281	8032	5353	5139	61794	32610	18339	12223	11734
0.70	19882	10491	5900	3932	3774	45398	23956	13471	8978	8618
0.80	15221	8032	4516	3009	2889	34755	18339	10312	6871	6596
0.90	12026	6345	3567	2377	2282	27459	14488	8146	5427	5210
1.00	9740	5139	2889	1925	1847	22240	11734	6596	4395	4218
1.50	4327	2282	1282	853	819	9880	5210	2927	1948	1870
2.00	2432	1282	719	478	459	5553	2927	1642	1092	1048
2.50	1555	819	459	305	292	3551	1870	1048	696	667
3.00	1079	567	317	210	202	2463	1296	725	480	461
3.50	792	416	232	153	147	1807	950	530	350	336

Abbreviation: SBP, systolic blood pressure; SNP, single nucleotide polymorphism. Calculations are based on SBP mean 98.57 and standard deviation 10.86.

Supplementary Table S6: Number of cases per 10 controls to detect significant association between the six SNPs and IR with statistical power of 80% and two-sided p-values of 0.05 (unadjusted) and 6.9 x 10^{-4} (adjusted for multiple testing) across a range of beta coefficients and allele frequencies for risk allele

OR	P=0.05					P=6.9 x 10^{-4}				
	Minor allele frequency					Minor allele frequency				
	0.05	0.10	0.20	0.40	0.50	0.05	0.10	0.20	0.40	0.50
1.10	9568	5076	2885	1963	1903	21865	11601	6593	4485	4345
1.20	2513	1340	769	533	522	5743	3063	1758	1219	1191
1.30	1171	628	364	257	253	2677	1435	832	586	578
1.40	690	372	217	156	155	1576	849	497	356	353
1.50	461	250	148	107	107	1055	571	337	245	245
1.60	335	182	108	80	80	765	416	248	183	183
1.70	256	140	84	63	63	586	320	193	144	145
1.80	204	112	64	51	52	467	257	156	117	119
1.90	168	93	57	43	44	384	212	130	99	100
2.00	142	79	48	37	38	324	180	111	85	87
2.10	122	68	42	33	33	278	155	96	75	76
2.20	106	59	37	29	30	242	136	85	66	68
2.30	94	53	33	26	27	214	121	76	60	61
2.40	84	47	30	24	24	191	108	69	55	56
2.50	76	43	27	22	22	173	98	63	50	51
3.00	50	29	19	16	16	115	67	44	36	37
3.50	37	22	15	12	13	85	51	34	29	29

Abbreviation: IR, insulin resistance; SNP, single nucleotide polymorphism. Calculations are based on 11% baseline risk for IR.

Supplementary Table S7: Correlation table for continuous cardio-metabolic traits showing Pearson's correlation coefficient and associated p-values

	BMI	SDS-BMI	WHR	SDS-WHR	SBP	SDS-SBP	DBP	SDS-DBP
**BMI**	1	0.86 (0.00)	0.48 ($1.00x10^{-13}$)	-0.10 ($1.46x10^{-4}$)	0.40 (0.00)	0.22 ($1.00x10^{-13}$)	0.29 ($1.00x10^{-13}$)	0.14 ($2.42x10^{-7}$)
**SDS-BMI**	0.86 (0.00)	1	0.54 ($1.00x10^{-13}$)	0.17 ($1.19x10^{-10}$)	0.28 (0.00)	0.22 ($1.02x10^{-13}$)	0.20 ($1.06x10^{-13}$)	0.15 ($7.04x10^{-13}$)
**WHR**	0.48 ($1.00x10^{-13}$)	0.54 ($1.00x10^{-13}$)	1	0.43 ($1.00x10^{-13}$)	0.08 ($2.00x10^{-3}$)	0.13 ($2.93x10^{-6}$)	0.04 (0.09)	0.09 ($2.00x10^{-3}$)
**SDS-WHR**	-0.10 ($1.46x10^{-4}$)	0.17 ($1.19x10^{-10}$)	0.43 ($1.00x10^{-13}$)	1	-0.26 ($1.00x10^{-13}$)	0.03 (0.28)	-0.15 ($3.80x10^{-8}$)	0.07 (0.01)
**SBP**	0.40 ($1.00x10^{-13}$)	0.28 ($1.00x10^{-13}$)	0.08 ($2.00x10^{-3}$)	-0.26 ($1.00x10^{-13}$)	1	0.92 (0.00)	0.63 ($1.00x10^{-13}$)	0.53 ($1.00x10^{-13}$)
**SDS-SBP**	0.22 ($1.00x10^{-13}$)	0.22 ($1.02x10^{-13}$)	0.13 ($3.00x10^{-6}$)	0.03 (0.28)	0.92 (0.00)	1	0.57 ($1.00x10^{-13}$)	0.62 ($1.00x10^{-13}$)
**DBP**	0.29 ($1.00x10^{-13}$)	0.20 ($1.06x10^{-13}$)	0.04 (0.09)	-0.15 ($3.79x10^{-8}$)	0.63 ($1.00x10^{-13}$)	0.57 ($1.00x10^{-13}$)	1	0.96 (0.00)
**SDS-DBP**	0.14 ($2.42x10^{-7}$)	0.15 ($7.04x10^{-8}$)	0.09 ($2.00x10^{-3}$)	0.07 (0.01)	0.53 ($1.00x10^{-13}$)	0.62 ($1.00x10^{-13}$)	0.96 (0.00)	1
**LDL**	0.18 ($1.75x10^{-11}$)	0.20 ($1.25x10^{-13}$)	0.16 ($4.06x10^{-10}$)	0.06 (0.03)	0.11 ($2.40x10^{-5}$)	0.12 (0.00)	0.07 (0.01)	0.07 (0.01)
**HDL**	-0.36 ($1.00x10^{-13}$)	-0.30 ($1.00x10^{-13}$)	-0.20 ($1.17x10^{-13}$)	-0.07 (0.01)	-0.08 ($2.00x10^{-3}$)	-0.01 (0.62)	-0.05 (0.07)	$1.00x10^{-3}$ (0.97)
**TC**	0.08 ($4.00x10^{-3}$)	0.10 ($1.42x10^{-4}$)	0.09 ($1.00x10^{-3}$)	0.02 (0.40)	0.11 ($4.80x10^{-5}$)	0.14 ($7.05x10^{-7}$)	0.08 ($3.00x10^{-3}$)	0.10 ($1.00x10^{-3}$)
**TG**	0.46 ($1.00x10^{-13}$)	0.40 ($1.00x10^{-13}$)	0.27 (0.00)	-0.02 (0.50)	0.22 ($1.00x10^{-13}$)	0.16 ($1.54x10^{-8}$)	0.13 ($7.63x10^{-7}$)	0.07 ($9.00x10^{-3}$)
**FG**	0.13 ($1.00x10^{-6}$)	0.09 ($1.00x10^{-3}$)	0.04 (0.11)	-0.16 (0.00)	0.18 ($1.08x10^{-11}$)	0.11 ($4.00x10^{-4}$)	0.09 ($1.00x10^{-3}$)	0.05 (0.09)
**FI**	0.62 ($1.00x10^{-13}$)	0.46 ($1.00x10^{-13}$)	0.29 ($1.00x10^{-13}$)	-0.07 (0.02)	0.28 ($1.00x10^{-13}$)	0.16 ($5.80x10^{-7}$)	0.19 ($1.85x10^{-10}$)	0.09 ($6.00x10^{-3}$)
**HOMA-IR**	0.61 ($1.00x10^{-13}$)	0.44 ($1.00x10^{-13}$)	0.28 ($1.00x10^{-13}$)	-0.08 ($6.00x10^{-3}$)	0.29 ($1.00x10^{-13}$)	0.17 ($9.20x10^{-8}$)	0.19 ($4.51x10^{-11}$)	0.09 ($3.00x10^{-3}$)
**HOMA-B**	0.56 ($1.00x10^{-13}$)	0.40 ($1.00x10^{-13}$)	0.25 ($1.00x10^{-13}$)	-0.05 (0.13)	0.23 ($1.00x10^{-13}$)	0.13 ($2.70x10^{-5}$)	0.15 ($2.44x10^{-7}$)	0.06 (0.04)

Abbreviations: BMI, body mass index; FG, fasting glucose; FI, fasting insulin; HDL, high density lipoprotein cholesterol; HOMA-IR, homeostatic model assessment of insulin resistance; HOMA-B, homeostatic model assessment of beta cell function; LDL, low density lipoprotein cholesterol; SDS, standard deviation scores; TC, total cholesterol; TG, triglycerides. Data are Pearson's correlation coefficient (p-value). P-values of 0.00 indicate that the value is $< 1.00 \times 10^{-36}$.

Supplementary Table S7 Continued: Correlation table for continuous cardio-metabolic traits showing Pearson's correlation coefficient and associated p-values

	LDL	HDL	TC	TG	FG	FI	HOMA-IR	HOMA-B
**BMI**	0.18 ($1.75x10^{-11}$)	-0.36 ($1.00x10^{-13}$)	0.08 ($4.00x10^{-3}$)	0.46 ($1.00x10^{-13}$)	0.13 ($1.00x10^{-6}$)	0.62 ($1.00x10^{-13}$)	0.61 ($1.00x10^{-13}$)	0..53 ($1.00x10^{-13}$)
**SDS-BMI**	0.20 ($1.25x10^{-13}$)	-0.30 ($1.00x10^{-13}$)	0.10 ($1.42x10^{-4}$)	0.40 ($1.00x10^{-13}$)	0.09 ($1.00x10^{-3}$)	0.46 ($1.00x10^{-13}$)	0.44 ($1.00x10^{-13}$)	0.40 ($1.00x10^{-13}$)
**WHR**	0.16 ($4.06x10^{-10}$)	-0.20 ($1.17x0^{-13}$)	0.09 ($1.00x10^{-3}$)	0.27 ($1.00x10^{-13}$)	0.04 (0.11)	0.29 ($1.00x10^{-13}$)	0.28 ($1.00x10^{-13}$)	0.25 ($1.00x10^{-13}$)
**SDS-WHR**	0.06 (0.03)	-0.07 (0.01)	0.02 (0.40)	-0.02 (0.50)	-0.16 ($1.01x10^{-9}$)	-0.07 (0.02)	-0.08 ($6.00x10^{-3}$)	-0.05 (0.13)
**SBP**	0.11 ($2.40x10^{-5}$)	-0.08 ($2.00x10^{-3}$)	0.11 ($4.80x10^{-5}$)	0.22 ($1.00x10^{-13}$)	0.18 ($1.08x10^{-11}$)	0.28 ($1.00x10^{-13}$)	0.29 ($1.00x10^{-13}$)	0.23 ($1.00x10^{-13}$)
**SDS-SBP**	0.12 ($1.00x10^{-5}$)	-0.01 (0.62)	0.14 ($7.05x10^{-7}$)	0.16 ($1.54x10^{-8}$)	0.11 ($1.00x10^{-4}$)	0.16 ($1.00x10^{-6}$)	0.17 ($9.20x10^{-8}$)	0.13 ($2.68x10^{-5}$)
**DBP**	0.07 (0.01)	-0.05 (0.07)	0.08 ($3.00x10^{-3}$)	0.13 ($7.63x10^{-7}$)	0.09 ($1.00x10^{-3}$)	0.19 ($1.85x10^{-10}$)	0.19 ($4.51x10^{-11}$)	0.15 ($2.44x10^{-7}$)
**SDS-DBP**	0.07 (0.01)	$1.00x10^{-3}$ (0.97)	0.10 ($1.00x10^{-3}$)	0.07 ($9.00x10^{-3}$)	0.05 (0.09)	0.09 ($6.00x10^{-3}$)	0.09 ($3.00x10^{-3}$)	0.06 (0.04)
**LDL**	1	0.10 ($8.10x10^{-5}$)	0.89 (0.00)	0.42 ($1.00x10^{-13}$)	0.28 ($1.00x10^{-13}$)	0.08 ($5.00x10^{-3}$)	0.12 ($6.20x10^{-5}$)	0.03 (0.27)
**HDL**	0.10 ($8.10x10^{-5}$)	1	0.34 ($1.00x10^{-13}$)	-0.37 ($1.00x10^{-13}$)	0.18 ($3.61x10^{-12}$)	-0.32 ($1.00x10^{-13}$)	-0.28 ($1.00x10^{-13}$)	-0.33 ($1.00x10^{-13}$)
**TC**	0.89 (0.00)	0.34 ($1.00x10^{-13}$)	1	0.38 (0.00)	0.34 ($1.00x10^{-13}$)	-0.02 (0.55)	0.03 (0.28)	-0.08 ($6.00x10^{-3}$)
**TG**	0.42 ($1.00x10^{-13}$)	-0.37 ($1.00x10^{-13}$)	0.38 ($1.00x10^{-13}$)	1	0.20 ($1.03x10^{-13}$)	0.41 ($1.00x10^{-13}$)	0.42 ($1.00x10^{-13}$)	0.33 ($1.00x10^{-13}$)
**FG**	0.28 ($1.00x10^{-13}$)	0.18 ($3.61x10^{-12}$)	0.34 ($1.00x10^{-13}$)	0.20 ($1.03x10^{-13}$)	1	0.16 ($6.49x10^{-8}$)	0.29 ($1.00x10^{-13}$)	$4.00x10^{-4}$ (0.99)
**FI**	0.08 ($5.00x10^{-3}$)	-0.32 ($1.00x10^{-13}$)	-0.02 (0.55)	0.41 ($1.00x10^{-13}$)	0.16 ($6.49x10^{-8}$)	1	0.98 (0.00)	0.98 (0.00)
**HOMA-IR**	0.12 ($6.20x10^{-5}$)	-0.28 ($1.00x10^{-13}$)	0.03 (0.28)	0.42 ($1.00x10^{-13}$)	0.29 ($1.00x10^{-13}$)	0.98 (0.00)	1	0.94 (0.00)
**HOMA-B**	0.03 (0.27)	-0.33 ($1.00x10^{-13}$)	-0.08 ($6.00x10^{-3}$)	0.33 ($1.00x10^{-13}$)	$4.00x10^{-4}$ (0.99)	0.98 (0.00)	0.94 (0.00)	1

Abbreviations: BMI, body mass index; FG, fasting glucose; FI, fasting insulin; HDL, high density lipoprotein cholesterol; HOMA-IR, homeostatic model assessment of insulin resistance; HOMA-B, homeostatic model assessment of beta cell function; LDL, low density lipoprotein cholesterol; SDS, standard deviation scores; TC, total cholesterol; TG, triglycerides. Data are Pearson's correlation coefficient (p-value). P-values of 0.00 indicate that the value is $< 1.00 \times 10^{-36}$.

Supplementary Table S8: Shapiro-Wilk test for normality of continuous traits before and after inverse normal transformations

Trait	P-value before transformation	P-value after transformation
Adiponectin (μg/ml)	1.50×10^{-14}	6.21×10^{-1}
BMI (kg/m^2)	8.71×10^{-20}	1.00
WHR	2.92×10^{-7}	1.00
SBP (mmHg)	6.69×10^{-13}	8.93×10^{-10}
DBP (mmHg)	7.20×10^{-17}	8.43×10^{-14}
LDL Cholesterol (mg/dL)	5.66×10^{-12}	1.00
HDL Cholesterol (mg/dL)	6.67×10^{-8}	7.55×10^{-1}
Total cholesterol (mg/dL)	3.76×10^{-8}	1.00
Triglycerides (mg/dL)	1.93×10^{-32}	9.71×10^{-1}
Fasting glucose (mmol/L)	1.00×10^{-3}	3.02×10^{-1}
Fasting insulin (mIU/L)	5.20×10^{-36}	1.00
HOMA-IR	6.67×10^{-37}	1.00
HOMA-B	1.14×10^{-35}	1.00

Abbreviations: BMI, body mass index; DBP, diastolic blood pressure; HDL, high density lipoprotein cholesterol; HOMA-IR, homeostatic model assessment of insulin resistance; HOMA-B, homeostatic model assessment of beta cell function; LDL, low density lipoprotein cholesterol; SBP, systolic blood pressure; WHR, waist-to-hip ratio.

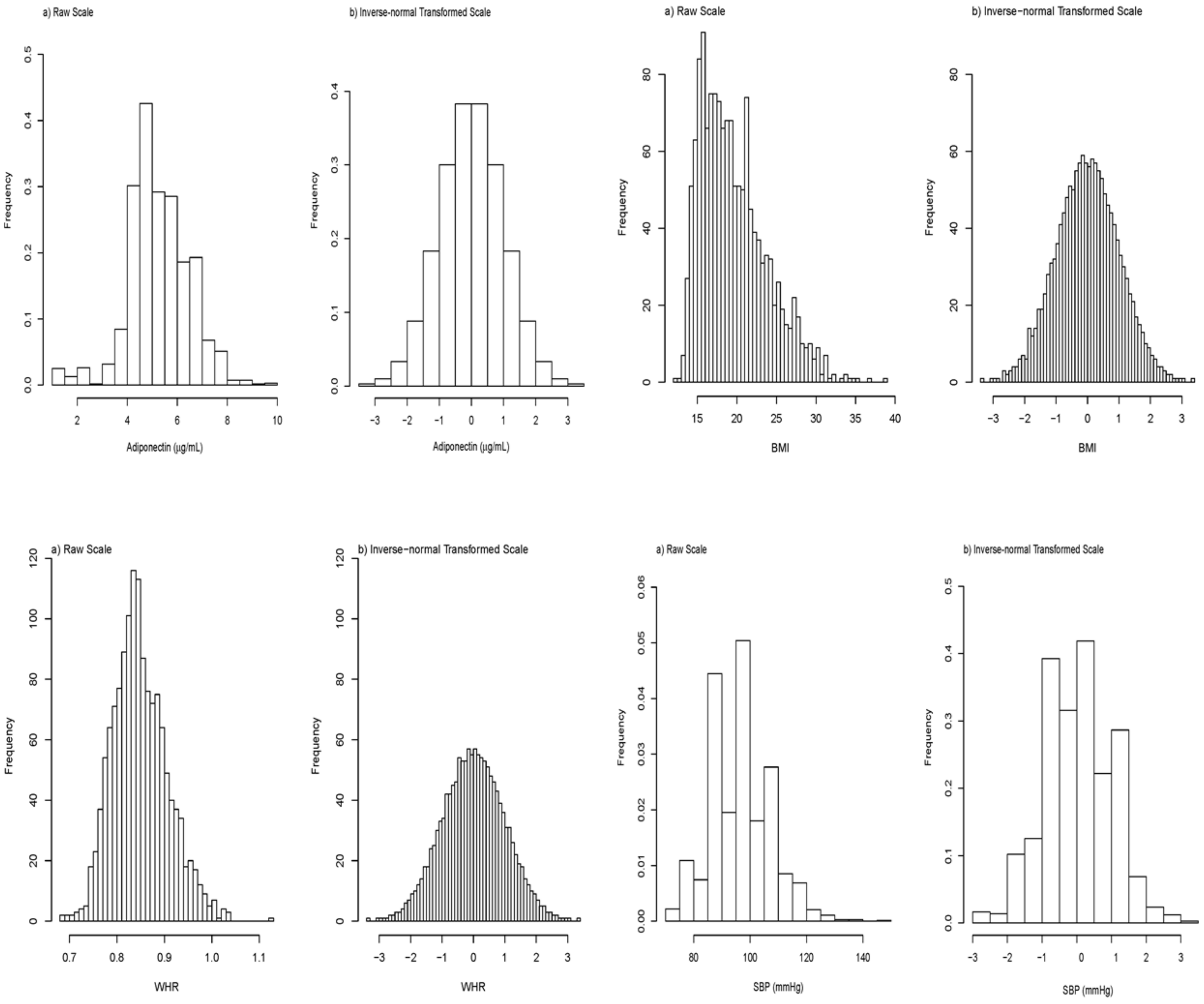

Supplementary Figure S1: Histograms illustrating raw distribution (panel A) and corrected distributions following inverse normal transformations (panel B) of variables of interest.

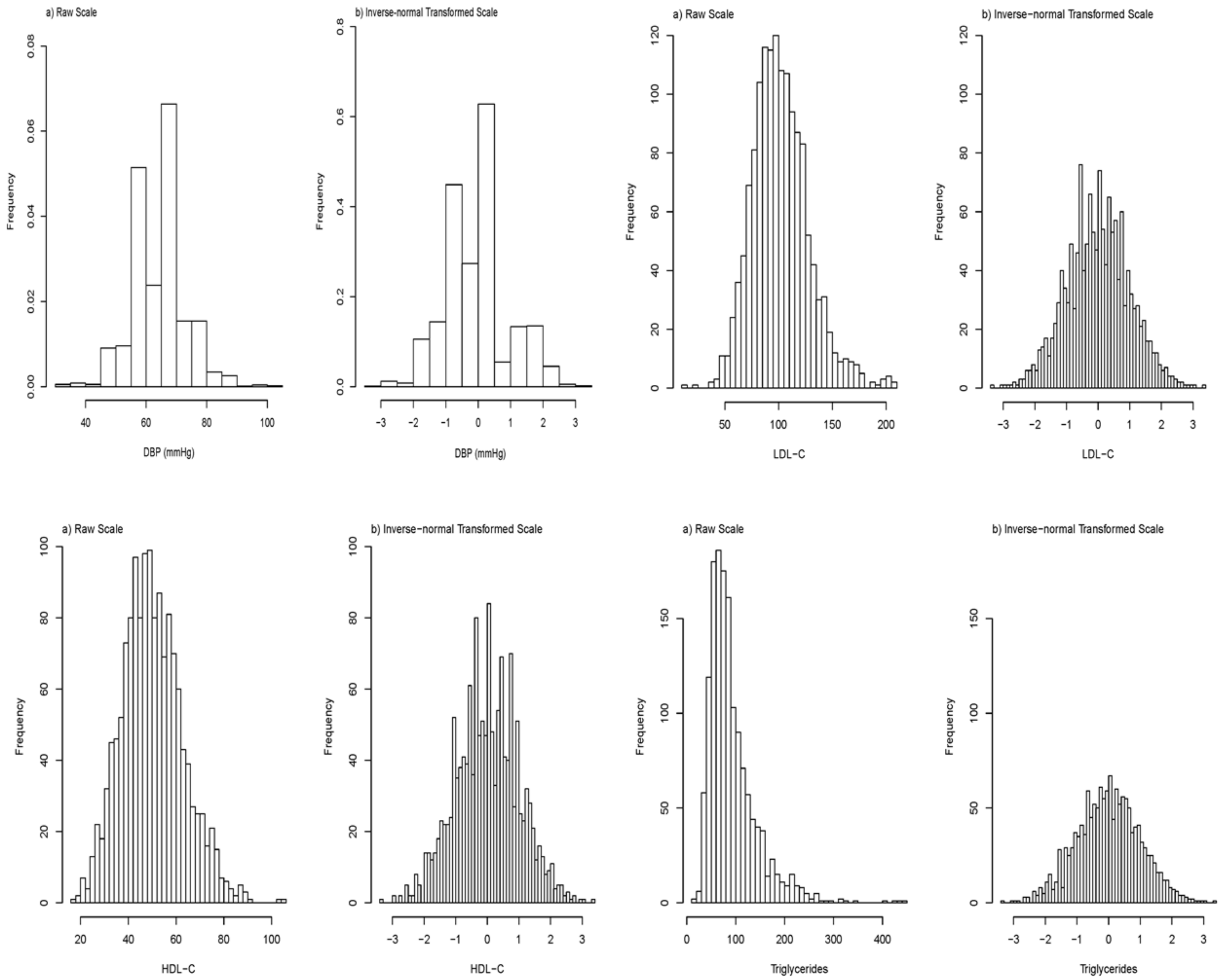

Supplementary Figure S1 Continued: Histograms illustrating raw distribution (panel A) and corrected distributions following inverse normal transformations (panel B) of variables of interest.

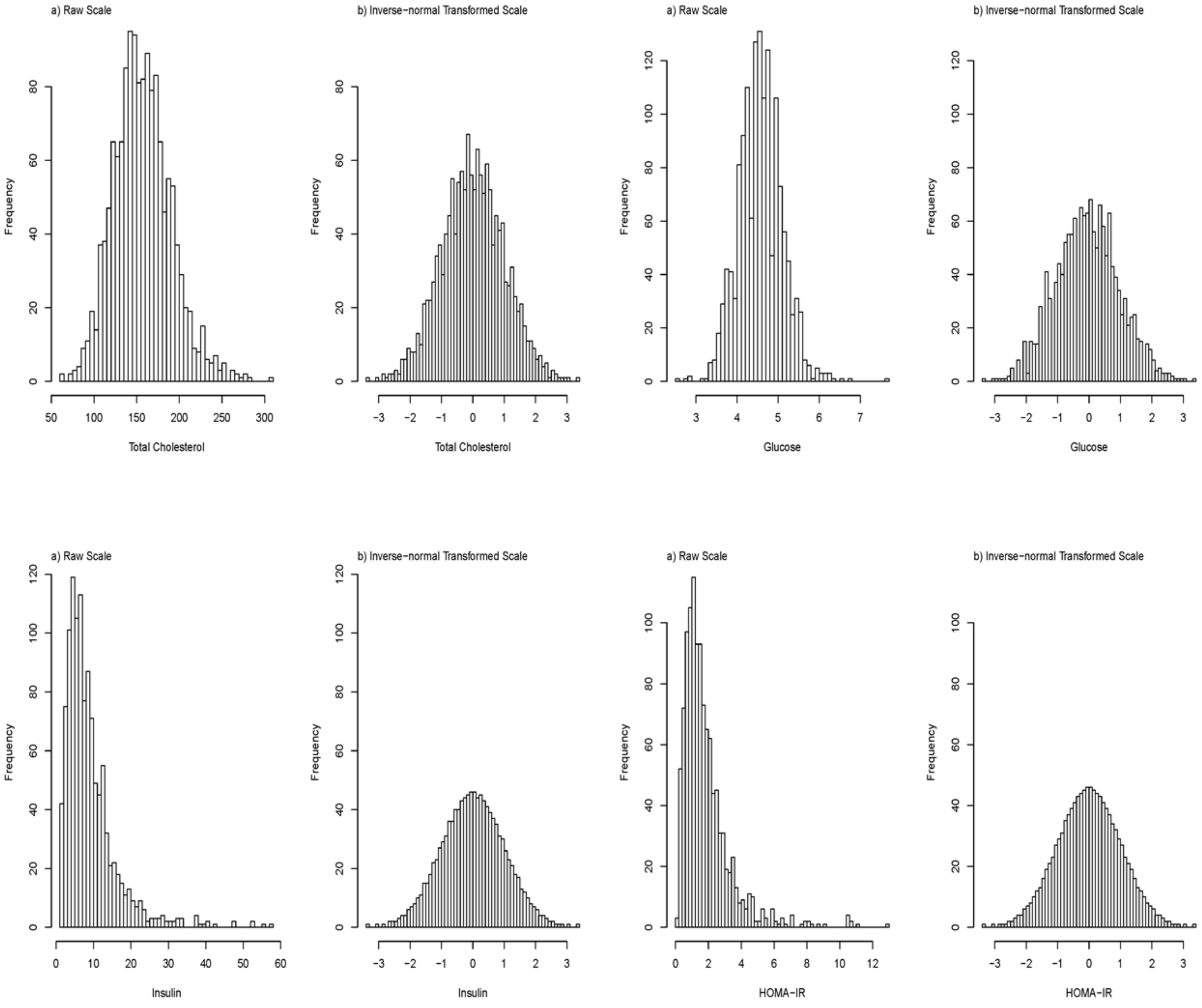

Supplementary Figure S1 Continued: Histograms illustrating raw distribution (panel A) and corrected distributions following inverse normal transformations (panel B) of variables of interest.

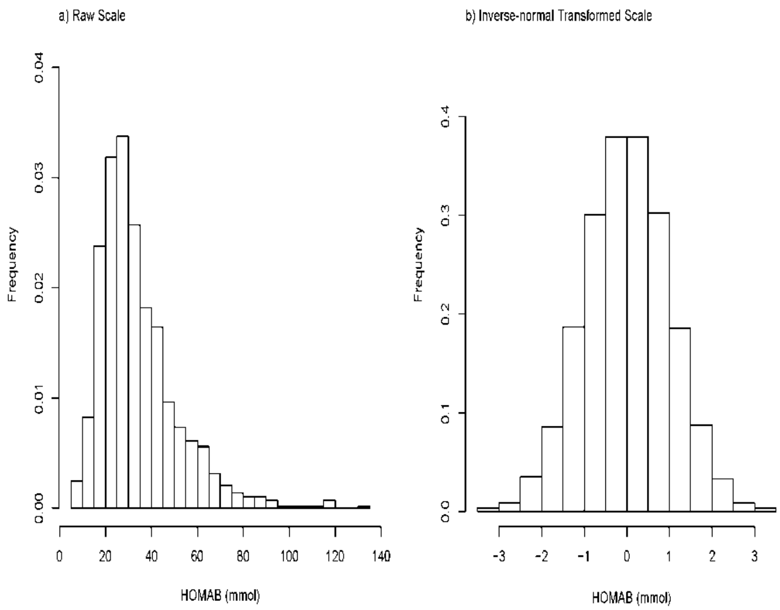

Supplementary Figure S1 Continued: Histograms illustrating raw distribution (panel A) and corrected distributions following inverse normal transformations (panel B) of variables of interest.

Supplementary Figure S1: Histograms illustrating raw distribution (panel A) and corrected distributions following inverse normal transformations (panel B) of variables of interest.
